# Epithelial SCAP/INSIG/SREBP Signaling Regulates Multiple Biological Processes during Perinatal Lung Maturation

**DOI:** 10.1371/journal.pone.0091376

**Published:** 2014-05-07

**Authors:** James P. Bridges, Angelica Schehr, Yanhua Wang, Liya Huo, Valérie Besnard, Machiko Ikegami, Jeffrey A. Whitsett, Yan Xu

**Affiliations:** 1 Perinatal Institute, Division of Pulmonary Biology, Cincinnati Children's Hospital Medical Center, Cincinnati, Ohio, United States of America; 2 INSERM U700, Paris, France; University of Tübingen, Germany

## Abstract

Pulmonary surfactant is required for lung function at birth and throughout postnatal life. Defects in the surfactant system are associated with common pulmonary disorders including neonatal respiratory distress syndrome and acute respiratory distress syndrome in children and adults. Lipogenesis is essential for the synthesis of pulmonary surfactant by type II epithelial cells lining the alveoli. This study sought to identify the role of pulmonary epithelial SREBP, a transcriptional regulator of cellular lipid homeostasis, during a critical time period of perinatal lung maturation in the mouse. Genome wide mRNA expression profiling of lung tissue from transgenic mice with epithelial-specific deletions of *Scap* (*Scap^Δ/Δ^*, resulting in inactivation of SREBP signaling) or *Insig1* and *Insig2* (*Insig1/2* ^Δ/Δ^, resulting in activation of SREBP signaling) was assessed. Differentially expressed genes responding to SREBP perturbations were identified and subjected to functional enrichment analysis, pathway mapping and literature mining to predict upstream regulators and transcriptional networks regulating surfactant lipid homeostasis. Through comprehensive data analysis and integration, time dependent effects of epithelial SCAP/INSIG/SREBP deletion and defined SCAP/INSIG/SREBP-associated genes, bioprocesses and downstream pathways were identified. SREBP signaling influences epithelial development, cell death and cell proliferation at E17.5, while primarily influencing surfactant physiology, lipid/sterol synthesis, and phospholipid transport after birth. SREBP signaling integrated with the Wnt/β-catenin and glucocorticoid receptor signaling pathways during perinatal lung maturation. SREBP regulates perinatal lung lipogenesis and maturation through multiple mechanisms by interactions with distinct sets of regulatory partners.

## Introduction

Pulmonary surfactant forms mono- and multilayer films that line the alveolar surface, reducing the surface tension created at the air-liquid interface to ensure proper inflation and function of the lung. Qualitative and quantitative defects in the surfactant system are associated with common pulmonary disorders including neonatal respiratory distress syndrome (RDS), bronchopulmonary dysplasia (BPD) and acute respiratory distress syndrome (ARDS) in children and adults. Mutations in genes critical for surfactant production or function, including surfactant proteins A (*SFTPA*), B (*SFTPB*) and C (*SFTPC*), and *ABCA3* are associated with chronic lung disease in humans and mice [1], .

Surfactant is a protein-lipid complex comprised of 90% lipid and 10% surfactant-associated proteins. Phospholipids represent 70–80% of the surfactant by weight, of which 45% is represented by disaturated phosphatidylcholine (SatPC) [3]. Transcriptional induction of pathways regulating lipid synthesis and surfactant proteins occurs during the later stages of perinatal lung maturation and is required for respiratory function at birth; thus, alveolar lipid and surfactant pool size requires precise regulation to ensure proper adaptation to the transition to air breathing.

Sterol regulatory element-binding proteins (SREBPs) are master transcriptional regulators of cellular lipid homeostasis active in multiple tissues. Three distinct SREBP isoforms, SREBP-1a, -1c and -2, are encoded by two genes (*Srebf1 and Srebf2*). In general, SREBP-1a and SREBP-1c regulate fatty acid synthesis while SREBP-2 regulates cholesterol synthesis [4], [5]. When intracellular cholesterol levels are abundant, SREBP proteins are held in an inactive form in the endoplasmic reticulum (ER) complexed to the sterol-sensing protein SCAP (SREBP cleavage-activating protein) and the ER-resident proteins INSIG1 and INSIG2. In sterol-depleted cells, INSIG1/2 dissociate from SCAP and the SREBP-SCAP complex is transported to the Golgi where the active form of SREBP is generated and released from the membrane by two sequential proteolytic cleavages by Site-1 and Site-2 proteases [6], [7]. The active form of SREBP enters the nucleus where it regulates transcription of target genes.

Lipogenesis is critical for the synthesis of pulmonary surfactant by type II cells. Phospholipid synthesis in type II cells was markedly increased shortly before birth in preparation of air breath. SREBP-1c is expressed in the developing lung and its expression increases during late gestation, in association with increased surfactant lipid synthesis [8]. SREBP-1c activates PCYT1A [9]–[11], the rate-limiting enzyme for phosphatidylcholine (PC) synthesis, thereby increasing PC synthesis in type II cells. Our recent studies showed that deletion of *Scap* in the adult mouse lung inhibited SREBP activity in respiratory epithelial cells, resulting in altered pulmonary lipid homeostasis associated with compensatory lipid synthesis and accumulation in lipofibroblasts [12]. Conversely, deletion of the *Insig1* and *Insig2* genes (*Insig1/2^Δ/Δ^*) activated SREBP-mediated lipogenesis in respiratory epithelial cells resulting in lipotoxicity-related lung inflammation and tissue remodeling in adult mice [13]. Transcriptional network modeling revealed that SREBP is an important regulatory hub in a lung lipid transcriptional network [14]. Collectively, data from both bioinformatic and mouse models demonstrated important roles for the SREBP signaling in lung lipid homeostasis.

This study was designed to further define the dynamic roles of SREBP in perinatal lung development using integrative approaches. Through comprehensive data analysis and integration, we identified early vs. late effects of epithelial SCAP/INSIG/SREBP signaling and defined SCAP/INSIG/SREBP-associated genes, bioprocesses and downstream pathways. We show that SREBP signaling influences epithelial development, cell death and cell proliferation at E17.5, while primarily influencing surfactant physiology, lipid/sterol synthesis, and phospholipid transport at PN1. In addition to regulation of the lipogenesis program that was dominant during the later stages of lung development, our study demonstrates clear cross-talk between the SCAP/INSIG/SREBP pathway and two other signaling pathways important for lung development and maturation, specifically Wnt/β-catenin and glucocorticoid receptor signaling.

## Materials and Methods

This study was carried out in accordance with the recommendations in the Guide for the Care and Use of Laboratory Animals of the National Institutes of Health. All study protocols were approved by the Institutional Animal Care and Use Committee of the Cincinnati Children's Hospital Research Foundation (Permit Number: 2C12114). All microarray data are MIAME compliant and submitted to GEO (http://www.ncbi.nlm.nih.gov/geo/).

### Transgenic Mice

*Scap^flox/flox^* mice containing a loxP-flanked neomycin resistance cassette located 3 kb upstream of exon 1 and an additional loxP site in intron 1 were obtained from Jackson Laboratory (Bar Harbor, ME). Epithelial-specific, conditional *Scap* knockout mice, termed *Scap^Δ/Δ^*, were generated crossing *Scap^flox/flox^* mice with: 1) mice that express rtTA protein in the respiratory epithelium (*SP-C-rtTA^−/tg^*); and 2) tetO-Cre recombinase mice (*(tetO)_7_CMV-Cre^tg/tg^*). This colony was maintained in a mixed FVB/N and B6/129S6 background.

*Scap^flox/flox^* littermates lacking either *rtTA* or *Cre* genes served as controls. Dams were administered doxycycline chow (625 mg/kg, Harlan Teklad, Madison, WI) from E6.5 to E12.5 to delete *Scap* from respiratory epithelial cells. Importantly, doxycycline was withdrawn at E12.5 to circumvent possible rtTA/Cre-mediated toxicity effects. Lung tissue was collected from pups at E17.5, E18.5 and PN1 (day of birth).

*Insig1^flox/flox^* mice have LoxP sites flanking exon 1 and *Insig2* knockout mice (Insig2^−/−^) were generated by replacing exons 2 and 3 with a neo cassette. *Insig1^flox/flox^ and Insig2^−/−^* mice were obatined from The Jackson Laboratory (Bar Harbor, ME). Doxycycline-exposed (E6.5-12.5) *SP-C-rtTA^+/tg^/*(*tetO*)_7_ *CMV-Cre^+/tg^/Insig1^flox/flox^/Insig2* ^−/−^ mice are termed *Insig1/2* ^Δ/Δ^ mice (*Insig1* was conditionally deleted in the *Insig2* ^−/−^ background). *Insig1^flox/flox^/Insig2* ^−/−^ littermates lacking either *SP-C-rtTA* or *(tetO)_7_CMV-Cre* served as controls. Genotypes were identified by PCR from genomic tail DNA as described previously [15], [16].

Mice were maintained in a pathogen free environment under the supervision of the Institutional Animal Care and Use Committee of Cincinnati Children's Hospital Research Foundation. Mice were kept on a 14-10 light-dark cycle and were allowed access to food and water *ad libitum*.

### Tissue Collection

E17.5 and E18.5 mice were delivered by Cesarean section with anesthesia followed by exsanguination. PN1 mice were taken on the day of birth and euthanized with pentobarbital followed by exsanguination. Lungs were flash frozen for analysis.

### Cell Transfection

MLE15 cells were cultured in HITES media [17]. For study, 5×10^5^ cells were plated in 6-well plates the day before transfection. Cells were transfected with 30 pmol/well of si*Scap* (Silencer Select, Life Technologies Corporation) using Lipofectamine RNAiMAX Reagents (Life Technologies Corporation) according to the manufacturers protocol. Cells were harvested 48 hr post-transfection for RNA isolation and qPCR analysis.

### RNA Isolation and qPCR

Total RNA was isolated from whole lung homogenates using the RNeasy Mini Kit (Qiagen, Valencia, CA) and reverse transcribed using the iScript kit (BioRad, Hercules, CA). miRNA was isolated using the miRNeasy Mini kit with on-column DNase digestion (Qiagen, Valencia, CA) and reverse transcribed using the Taqman MicroRNA Reverse Transcription Kit (Life Technologies, Grand Island, NY). Quantitative PCR was performed on a StepOnePlus Real Time PCR System (Applied Biosystems, Foster City, CA). Data for RNA analysis were normalized to 18 s and data for miRNA analysis were normalized to snoRNA202.

### Saturated Phosphatidylcholine (Sat PC) and Total Phospholipid Measurement

Total lipids were extracted from fetal lung tissue using chloroform and methanol according to the Bligh and Dyer method [18]. Sat PC was then isolated using osmium tetroxide and neutral alumina by the Mason, et. al. method [19]. Isolated SatPC and total phospholipid were then quantitated by phosphorus measurement as previously described [20].

### Cholesterol and Triglyceride Quantification

Total cholesterol, cholesterol esters and triglycerides were determined using the Cholesterol/Cholesterol Ester Quantification kit (EMD Chemicals, Inc) and the Triglyceride Quantification kit (BioVision) according to the manufacturer's protocol.

### Microarray Analysis

Total lung RNA was isolated from *Scap^Δ/Δ^*, *Insig1/2* ^Δ/Δ^, and respective control littermates at E17.5, E18.5 and PN1 using RNeasy Mini Kit (Qiagen, Valencia, CA). The cRNA was then hybridized to the Mouse Gene 1.0 ST Array (Affymetrix, Santa Clara, CA) according to the manufacturer's protocol. Three biological replicates were used for each condition. The RNA quality and quantity assessment, probe preparation, labeling, hybridization, and image scan were carried out in the Cincinnati Children's Hospital Medical Center Affymetrix Core using standard procedure. Data were further analyzed using GeneSpring GX 11.5 (Agilent Technologies, Santa Clara, CA). Differentially expressed genes in *Scap^Δ/Δ^* vs. control mice at different developmental time points were identified using two-way ANOVA (genotype and time dependent gene changes). Genes altered in *Insig1/2* ^Δ/Δ^ vs. control were identified using Student's *t*-test with the threshold of corrected P≤0.01 and fold change ≥1.5. Benjamini-Hochberg false discovery rate (FDR) was applied for multiple testing correction. The probe sets were pre-filtered by their expression range within 35–100 percentiles for at least 66% of samples to remove probes with extremely low expression signals in both mutant and control samples. The complete dataset has been submitted to the Gene Expression Omnibus (GEO) database with the accession number of GSE53397.

### miRNA Analysis

miRNA expression profile was determined by using GeneChip miRNA 3.0 Array (www.affymetrix.com). This array platform provides complete coverage of miRBase v17, snoRNA and scaRNA probes on the same array, and covers 153 organisms including human, mouse and rat. Lung samples from *Scap^Δ/Δ^* and control littermates at E18.5 (n = 3 per genotype) were used to isolate RNA using miRNeasy kit with on column DNase digestion (Qiagen, Valencia, CA). The Expression Console software (Affymetrix, Inc.) was used for data summarization, normalization, and quality control. The miRNAs with P<0.01 and fold changes >1.3 was defined as differentially expressed using BRB-ArrayTools Version: 4.2.1 (http://linus.nci.nih.gov/BRB-ArrayTools.html). miRNA target prediction and filtering were performed using miRNA Target Filter tool in IPA. miRNA and mRNA targets showing inverse expression patterns in response to *Scap* deletion at E18.5 were selected for further analysis (i.e. miRNA up, mRNA down OR miRNA down, mRNA up).

Differentially expressed genes at different lung maturation stages (i.e. E17.5, E18.5 and PN1) were subjected to Self-organizing Map (SOM) clustering, gene ontology analysis, promoter analysis, functional classification and pathway analysis as described previously [21], [22]. Gene Ontology and Pathway analysis were performed using publicly available web-based tool DAVID (database for annotation, visualization, and integrated discovery), ToppGene Suite (http://toppgene.cchmc.org/) and Ingenuity Pathway Analysis (IPA, Ingenuity).

Upstream regulator analysis uses Ingenuity software based on prior knowledge of expected effects between transcriptional regulators and their target genes pre-compiled in the Ingenuity Knowledge Base. The analysis examines how many known targets of each transcriptional regulator are present in the user's dataset, and also compares their direction of change in the experimental condition to what is expected from the literature. If the observed direction of change is positively correlated with the literature, then we can predict the transcriptional regulator is at activated state. For each potential transcriptional regulator, two statistical measures (overlap p-value and activation z-score) are computed. The p-value is calculated to rank upstream regulators based on significant overlap of the differentially expressed genes to the known targets regulated by a transcriptional regulator. The z-score is used to infer likely activation states of upstream regulators.

Promoter sequences (2 kb upstream of the transcriptional start site) were retrieved using Biomart (www.ensembl.org/biomart/martview). Multiple promoter analytic tools (Genomatix Matinspector, Clover, CisView, and Paint4) were used to identify enriched transcription factor binding sites in the promoter regions of genes in the cluster of interest. Cytoscape (http://cytoscapeweb.cytoscape.org/) was used to map associations between transcription factors matrix families and predicted target genes. PBGE database (http://research.cchmc.org/pbge/) was used to match the transcription factors matrix families to the transcription factors expressed in lung.

## Results

### The Effects of Epithelial Scap Deletion on Perinatal Lung Lipid Content

**Figure 1:**
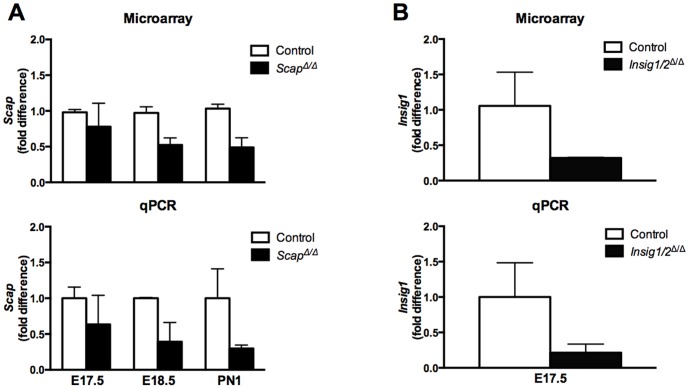
Relative expression of *Scap* and *Insig1* in *Scap^Δ/Δ^* and *Insig1/2^Δ/Δ^* mice. *Scap* (A) and *Insig1* (B) mRNA were measured by mRNA microarray analysis and qPCR. Total lung RNA was isolated from *Scap^Δ/Δ^*, *Insig1/2^Δ/Δ^* and littermate controls at E17.5, E18.5 and PN1. Data were normalized to 18S and presented as mean ± S.E.; n = 2–3/group.

**Figure 2:**
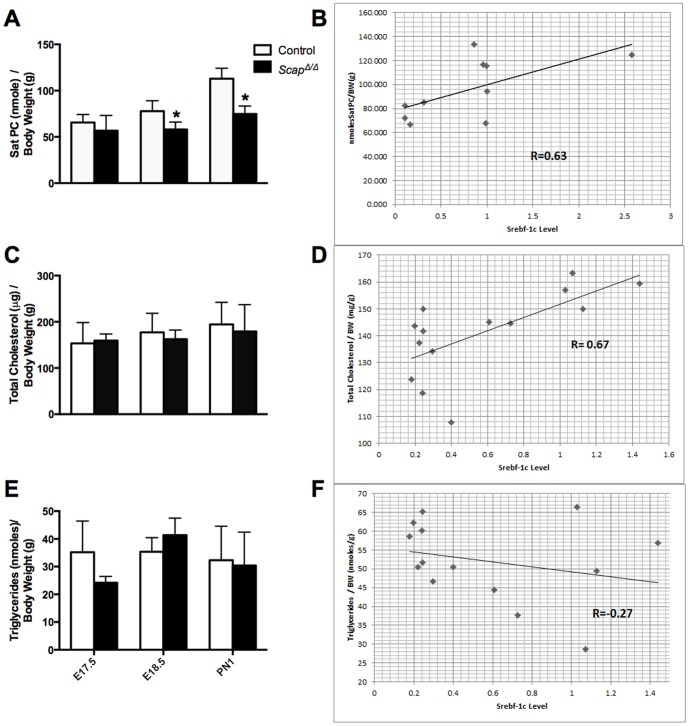
Altered lung surfactant SatPC content in *Scap^Δ/Δ^* mice. SatPC (A), total cholesterol (C) and triglycerides (E) were measured using whole lung samples from control (open bars) and *Scap^Δ/Δ^* mice (closed bars) at E17.5, E18.5 and PN1. *p<0.05. n = 5–8/group. Correlation coefficients between *Srebf-1c* mRNA and SatPC (B), cholesterol (D) and triglyceride levels (F) were measured at age PN1, n = 5–8/group.

Mice with conditional deletion of the *Scap* (*Scap^Δ/Δ^*) or *Insig1* (*Insig1/2* ^Δ/Δ^) genes in pulmonary epithelium were generated as previously reported [12], [13]. *Scap* and *Insig1* gene deletion efficiency was assessed by qPCR and exon deletion was confirmed by exon microarray, demonstrating a marked decrease in *Scap* (Figure 1A) and *Insig1* (Figure 1B) mRNA in our mouse models. We have previously demonstrated that epithelial SCAP/SREBP signaling regulates lipid homeostasis in the adult lung [12], [13]. To evaluate the effects of epithelial *Scap* gene deletion on perinatal lung lipid homeostasis, we measured lung SatPC, total cholesterol and triglyceride levels in lungs at E17.5, E18.5 and PN1. SatPC was significantly decreased at E18.5 and PN1 in *Scap^Δ/Δ^* mice vs. control littermates, but not at E17.5 (Fig. 2A). Changes in SatPC content correlated with the deletion efficiency of *Scap* in this model (Figure 1A). Both total cholesterol and triglyceride content were not significantly altered by group mean comparison between *Scap^Δ/Δ^* and control mice at any of the time points examined (Fig. 2C and E). Considering the sample variation related to the incomplete deletion efficiency of the *SP-C/tetO-Cre* transgenic system, we examined the correlation between *Srebf-1c* expression levels and lung lipid content at PN1. Results from this analysis showed that *Srebf-1c* levels positively correlated with SatPC and total cholesterol content, with correlation coefficients of 0.63 and 0.67, respectively; however, there was no strong correlation between *Srebf-1c* and triglyceride levels (−0.27) (Fig. 2B, D and F). These results indicated that, due to the variation of deletion efficiency and the possible compensatory effect of other cell types in the lung to the epithelial deletion of *Scap in vivo*, the t-test comparison of group mean may not be sensitive enough to reveal the direct effect of *Scap* deletion in epithelial cells on cholesterol levels. These data demonstrate that epithelial SCAP/SREBP signaling positively regulates SatPC content during perinatal lung maturation.

### The Functional Genomic Responses to SREBP Deletion/Activation during Perinatal Lung Maturation

**Figure 3:**
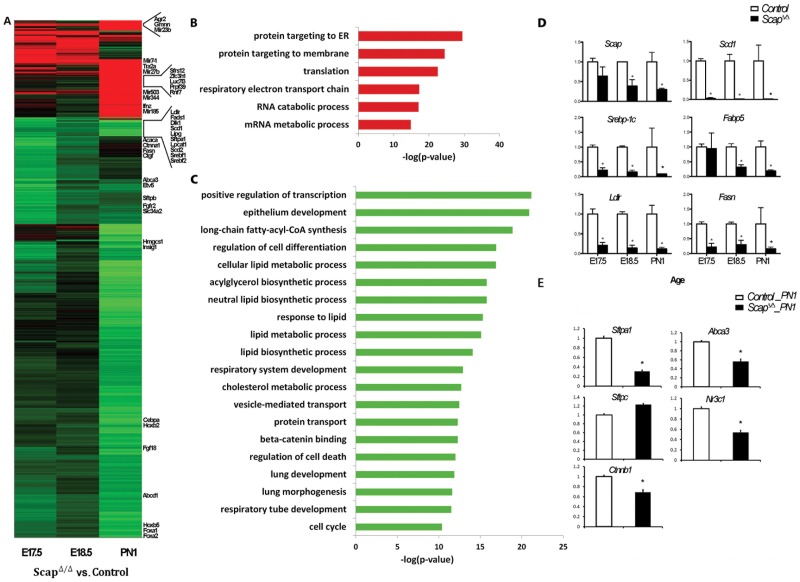
Genes and biological processes affected by *Scap* deletion in the developing lung. A) Differentially expressed genes in *Scap^Δ/Δ^* lungs were identified by mRNA microarray analysis. Heatmap analysis shows the two-dimensional clustering of 1079 mRNAs that were significantly altered in *Scap^Δ/Δ^* mice compared with controls. The intensity in the red and green color ranges indicates increased versus decreased mRNAs, respectively. Each row represents a single mRNA, and each column represents an average fold change between knockout and control mice at the specified gestational age. B and C) Biological processes significantly influenced by epithelial *Scap* deletion were identified using ToppGene Suite (http://toppgene.cchmc.org). Enriched bioprocesses in genes induced (B) or suppressed (C) in *Scap^Δ/Δ^* lungs are represented as red bars and green bars respectively. Statistical significance of each bioprocess was presented using negative log2 transformation of P value. D and E) qPCR analysis confirmed RNA microarray data demonstrating altered expression of genes involved in lipid biosynthesis/transport (D) and in lung development and maturation (E) in *Scap^Δ/Δ^* mice (filled bars) compared with control (open bars), n = 3/group, *p<0.05.

To systematically determine the genomic responses of SREBP deletion/activation in the perinatal lung, genome wide mRNA expression profiles of *Scap^Δ/Δ^* mouse lungs were performed at E17.5, E18.5 and PN1; *Insig1/2^Δ/Δ^* mouse lungs were analyzed at E17.5. Genes differentially expressed in *Scap^Δ/Δ^* mice, *Insig1/2^Δ/Δ^* mice and their corresponding control littermates in at least one of the conditions were identified by ANOVA. This analysis identified 184 genes that were up-regulated and 1079 genes down-regulated in *Scap^Δ/Δ^* mice, suggesting that SREBP works primarily as a transcriptional activator in the perinatal lung (Figure 3A and Table S1). Among the genes that increased in any of the three time points in *Scap^Δ/Δ^* lungs, “protein targeting to ER/membrane”, “RNA catabolic/metabolic process” were the most enriched functional categories (Figure 3B). Among the genes that were suppressed in *Scap^Δ/Δ^* lungs, “regulation of transcription”, “epithelium development”, “vesicle mediated transport/sorting”, “lung development”, “lipid biosynthetic/metabolic process”, and “regulation of cell death/cell cycle” were most enriched functional classes (Figure 3C).

We further assessed the functional enrichment of genes altered at E17.5 (early effect) and PN1 (later effect) separately to identify the dynamic effects of SREBP deletion/activation on perinatal lung development and maturation. Genes involved in “Cellular movement”, “Cardiovascular System Development and Function”, “Respiratory System Development and Function”, “Cellular Assembly and Organization”, “Cell Morphology”, “Cell Cycle” were the most highly enriched bioprocesses at E17.5, decreasing with time in response to disruption of SREBP signaling. In contrast, genes involved in “Lipid/Sterol Synthesis/Metabolism”, “Angiogenesis”, “Epithelial development/differentiation”, were mostly highly enriched at PN1 (Table 1). Canonical pathways significantly affected at E17.5 by *Scap* deletion include “Oxidative Phosphorylation”, “Mitochondrial Dysfunction”, “Wnt/β-catenin Signaling”, and “Glucocorticoid Receptor Signaling”; at the PN1 time point, pathways that were enriched included “Steroid Biosynthesis”, “Ephrin Receptor Signaling”, “Integrin Signaling” and “LXR/RXR Activation” (Table 2). Many of the known targets regulated by SREBP signaling were decreased in *Scap^Δ/Δ^* mice and/or induced in *Insig1/2^Δ/Δ^* mice (Table S2), including *Scd1*, *Scd2*, *Fasn*, *Ldlr*, *Hmgcs1*, *Sqle* and *Lss*, demonstrating an inverse relationship between SREBP suppression and activation in *Scap^Δ/Δ^* and *Insig1/2* ^Δ/Δ^ mice, respectively. In addition to the known effects of SREBP signaling on lipid metabolism that was observed predominantly at PN1, genes (*Sftpa1*, *Sftpb*, *Abca3*, *Ctnn1b* and *Nr3c1*) and pathways (Wnt/β-catenin Signaling) important in fetal lung development, maturation and function were decreased in *Scap^Δ/Δ^* mice at E17.5. qPCR validation of a subset of these microarray data are shown in Figure 3D and 3E. Collectively, these data demonstrate dynamic roles of epithelial SREBP signaling during perinatal lung development.

**Table 1:** Enriched functions altered by *Scap* deletion in perinatal lung.

E17.5 *Scap^Δ/Δ^*		E18.5 & PN1 *Scap^Δ/Δ^*	
Functions	p value	Functions	p value
Cellular Movement	2.79E-27	Synthesis of sterol	1.29E-14
Cardiovascular System Development and Function	8.96E-24	Synthesis of lipid	3.16E-12
Cellular Assembly and Organization	2.27E-23	Synthesis/metaboilsm of cholesterol	1.01E-11
Cellular Function and Maintenance	2.27E-23	Regulation of transcription	3.90E-11
Tissue Morphology	7.71E-23	Proliferation of cells	1.28E-08
Cellular Growth and Proliferation	1.33E-22	Cell death	1.83E-08
Cell Death	1.49E-15	Differentiation	9.57E-07
Cell Morphology	6.04E-15	Angiogenesis	1.13E-06
Respiratory System Development and Function	1.83E-13	Development of epithelial tissue	3.57E-06
Cell Cycle	1.52E-10	Fatty acid metabolism	4.09E-06

**Table 2:** Enriched canonical pathways altered by *Scap* deletion in perinatal lung.

E17.5 *Scap^Δ/Δ^*		PN1 *Scap^Δ/Δ^*	
Pathways	p value	Pathways	p value
Oxidative Phosphorylation	3.16E-14	Biosynthesis of Steroids	5.89E-08
Mitochondrial Dysfunction	2.00E-13	Ephrin Receptor Signaling	3.31E-06
Axonal Guidance Signaling	6.31E-11	Integrin Signaling	1.00E-05
Ubiquinone Biosynthesis	3.98E-09	CXCR4 Signaling	8.51E-05
Wnt/β-catenin Signaling	1.91E-08	Wnt/β-catenin Signaling	9.33E-05
Paxillin Signaling	2.29E-08	IL-8 Signaling	1.38E-04
PTEN Signaling	1.78E-07	ILK Signaling	1.07E-04
Phospholipase C Signaling	2.40E-06	Insulin Receptor Signaling	3.80E-04
Notch Signaling	1.23E-05	mTOR Signaling	4.68E-04
Glucocorticoid Receptor Signaling	7.59E-04	LXR/RXR Activation	8.32E-04

**Figure 4:**
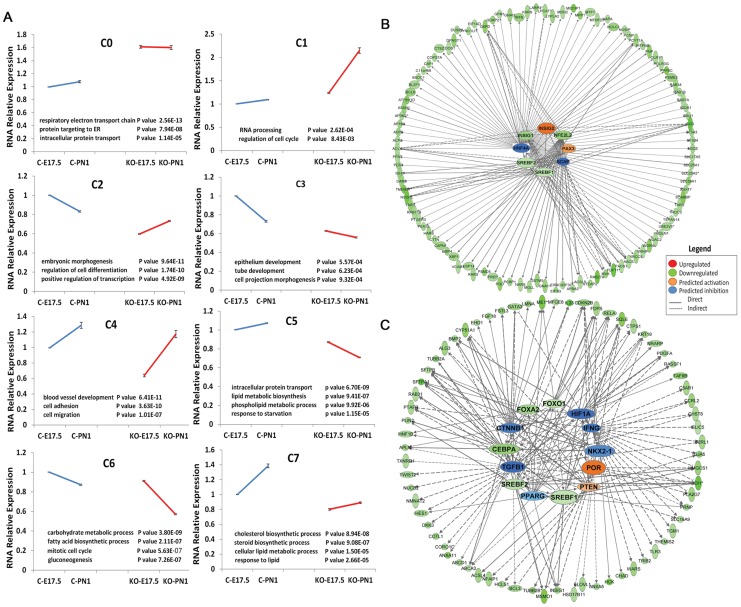
SREBP transcriptional network regulates lipogenic pathways. A) Clustering analysis of genes differentially expressed in *Scap^Δ/Δ^* mice vs. control at E17.5 and PN1 using a Self-organizing map. Genes from Clusters 5 and 7 were functionally enriched in lipid biosynthesis and metabolism. B) Upstream network analysis of Cluster 5 genes. Genes down-regulated in response to *Scap* deletion are shown as green nodes, genes that were unchanged but predicted to be involved in the process are shown in orange (activation) and blue (inhibition) respectively. C) Upstream network analysis of Cluster 7 genes. In addition to *Srebf1/2*, *Cebpa*, *Foxa2* and *Foxo1* were decreased in response to *Scap* deletion (shown as green nodes). Genes that were unaltered but predicted to be involved in the regulation of Cluster 7 genes, including *Por* and *Pten* (orange nodes indicate activation), *Tgfb1*, *Nkx2-1*, *Ifng*, *Pparg*, *Ctnnb1* and *Hif1a* (blue indicates inhibition), are shown.

In order to understand the regulatory mechanisms and identify potential cofactors of the SREBP signaling pathway in regulating lung lipid homeostasis, genes differentially expressed in *Scap^Δ/Δ^* mice vs. control from two-way ANOVA analysis was clustered into 8 different sub-groups based on expression pattern similarity using the SOM clustering method (Table S1, Figure 4A). Genes within each cluster were subjected to functional classification using Toppgene suite (http://toppgene.cchmc.org/). Gene set enrichment analysis identified two “lipid” enriched clusters, 5 and 7. Cluster 5 genes were functionally enriched in molecular transport (*Slc25a1*, *Slc11a1*, *Acsl5*), protein trafficking (*Arf6, Scamp2, Rab27a*), lipid/phospholipids biosynthesis and metabolism (*Pcyt1a, Lpcat1, Lypla2, Scd1, Aacs, Stx12*) (Figure 4B). Genes within this cluster were moderately induced before birth in control mice but decreased within the same time frame in *Scap^Δ/Δ^* mice (Figure 4A). Cluster 7 genes were induced with age in control mice but maintained at a low level from E17.5 to PN1 in *Scap^Δ/Δ^* mice. Genes within this cluster were functionally involved in cholesterol/steroid synthesis and in response to lipid (*Hmgcs1, Idl1, Sqle, Fdps, Msmo1, Cdipt, Insig1, Dgat1*) (Figure 4C). An important functional difference between the genes regulated at E17.5 compared to PN1 (in addition to regulation of lipid homeostasis) was the finding that a number of genes in cluster 7 play important roles during lung morphogenesis, maturation and surfactant function including *Foxa2*, *Fgf18*, *Sftpa1*, *Sftpb* and *Abca3*.

**Figure 5:**
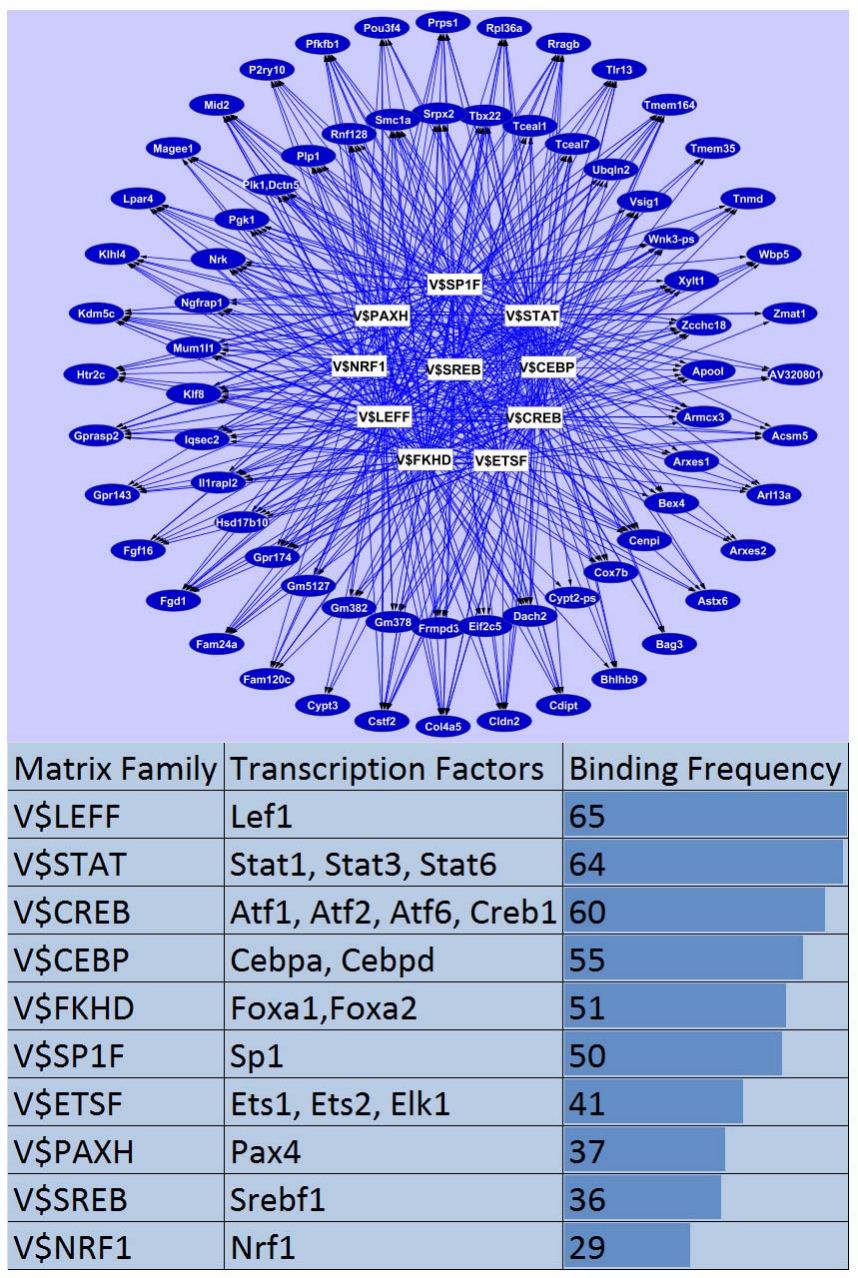
Putative SREBP co-factors mediating perinatal lung lipid homeostasis. A SREBP related transcriptional network was constructed based on the promoter analysis of genes within Clusters 5 and 7. Each connection between a matrix family (white rectangle) and a target gene (blue oval) represents at least one predicted binding site for a given transcription factor on the promoter region of that target gene.

To understand the role of SCAP/INSIG/SREBP signaling in the regulation of lung lipid homeostasis at a network level we sought to identify potential upstream regulators and downstream targets using promoter and literature mining approaches. Proximal promoter sequences of genes in cluster 5 and 7 (2 kb upstream of each gene) were extracted and promoter analysis was performed to identify significantly over-represented cis-elements and corresponding transcription factors that are expressed in lung. A transcriptional network was constructed using the top 10 ranked transcription factor binding sites and their potential target genes (genes that contain positive binding sites in promoter region) in Cytoscape (http://www.cytoscape.org/). As shown in Figure 5, transcription factors in the *Lef*, *Stat*, *Cebp*, *Ebox/Creb*, *Fkhd*, *SP1*, *Pax* and *Nrf1* families are important regulatory hubs in the network and likely to be partners of SREBP that regulate perinatal lung lipid homeostasis. Literature mining was performed using IPA knowledge base (https://analysis.ingenuity.com/). Potential upstream regulators were predicted using genes from cluster 5 and 7 respectively. The biological relationship of predicted core regulators and their associated targets (based on literature) are illustrated in Figure 4B&C. As expected, SCAP/INSIG/SREBP were predicted as core regulators for both cluster 5 and 7 genes, but other core regulators in the network were distinct, indicating multiple gene-specific regulatory mechanisms are involved.

### Identification of Potential Upstream Regulators of SREBP Signaling in Perinatal Lung Development

Differentially expressed genes in *Scap^Δ/Δ^* mice were subjected to an upstream regulator analysis to identify potential mediators that drive the overall genomic responses caused by deletion or activation of SREBP signaling. From this analysis, we identified potential regulators of SREBP signaling at different time points during lung maturation. Table 3 lists the top 10 candidate regulators that are most likely to be involved in the SREBP signaling pathway at E17.5, E18.5 and PN1, ranked by p-value. A group of transcriptional factors (TFs) including *Foxo3, Irf7, E2f4, Ctnnb1, Pou4f1, Gli1, and Gata3 (Gata6 may share similar group of downstream targets)* were predicted to regulate transcriptional responses to both the deletion of *Insig* and *Scap* at E17.5, but not at E18.5 or PN1. These transcription factors are known to positively regulate cell morphogenesis and proliferation, and negatively regulate cellular metabolic and biosynthetic processes, and programmed cell death. At E18.5 and PN1, *SREBF1/2*, *Foxo4*, *Nr1h2/3*, *Ppara* and *Pparg* were predicted to be important regulators of the SREBP signaling. The activation status, either activated or inhibited, of the predicted upstream regulators in *Scap^Δ/Δ^* and *Insig1/2* ^Δ/Δ^ mice are listed in Table 4. The finding that *Srebf1* and *2* are two of the most inhibited TFs (associated with the lowest z-scores) in *Scap^Δ/Δ^* mice, and the most activated TFs in *Insig1/2* ^Δ/Δ^ mice, demonstrates the efficacy of this method to predict novel partners, co-factors and signaling molecules influenced by SREBP signaling in the perinatal lung. In addition to *Srebf1* and *2*, the activation status of *Pparg*, *Nr1h*, *Myc* and *Cdkn2A* were inversely predicted in *Scap^Δ/Δ^* and *Insig1/2* ^Δ/Δ^ mice, indicating that these factors are likely to be proximate downstream regulators of the SCAP/INSIG/SREBP cascade. The activation status of *Cebpa*, *Ahr*, *Irf1*, *Nrf1* and *Hnf4a* were predicted to be in the same direction for *Scap^Δ/Δ^* and *Insig1/2* ^Δ/Δ^ mice, suggesting that these factors are likely influencing or co-regulating SCAP/SREBP signaling by more than one mechanism.

**Table 3:** Potential upstream regulators of SCAP/SREBP pathway (ranked by P-value).

E17.5 Only		E17.5 Insig^Δ/Δ^		E17.5 Scap^Δ/Δ^		E18.5 Scap^Δ/Δ^		PN1 Scap^Δ/Δ^	
Gene	P value	Gene	P value	Gene	P value	Gene	P value	Gene	P value
FOXO3	0.000599	SREBF2	2.44E-09	WT1	5.33E-06	SREBF2	3.26E-06	SREBF2	1.02E-12
IRF7	0.000997	TRIM24	2.07E-08	SPDEF	7.2E-06	FOXO4	4.17E-06	SREBF1	3.51E-10
E2F4	0.00373	SIRT2	5.33E-06	PROX1	2.32E-05	SREBF1	1.85E-05	SIRT2	3.59E-10
CTNNB1	0.00663	TP53	1.76E-05	KLF2	7.18E-05	NR1H3	0.000022	FOXO4	4.6E-07
POU4F1	0.00881	PPARA	0.000106	HHEX	9.65E-05	SIRT2	3.64E-05	PPARA	2.49E-05
GLI1	0.00989	WT1	0.000106	NFE2L2	0.000435	NR1H2	0.000452	NR1H2	7.24E-05
GATA3	0.0231	STAT3	0.00018	NOTCH1	0.000699	STAT6	0.000464	PPARGC1B	0.000173
SPDEF	7.2E-06	Smad2/3/4	0.000599	HTT	0.000829	PPARGC1B	0.00071	NR1H3	0.000189
		TOB1	0.000724	NFkB	0.000905	PPARA	0.00305	PPARG	0.00088
		SREBF1	0.000742	IRF7	0.000997	ETS1	0.00485	ESRRA	0.0017

**Table 4:** Potential upstream regulators of SCAP/SREBP pathway (ranked by Z-score).

Insig^Δ/Δ^			Scap^Δ/Δ^		
Gene	State	Z-score	Gene	State	Z-score
IRF7	Activated	4.326	MYC	Activated	5.109
NFkB (complex)	Activated	4.1	MYCN	Activated	4.464
SREBF1	Activated	3.999	PPARGC1A	Activated	3.683
CDKN2A	Activated	3.805	SMAD7	Activated	3.407
STAT1	Activated	3.457	NRF1	Activated	3.283
SREBF2	Activated	3.375	SPDEF	Activated	3.099
IRF1	Activated	3.372	HIC1	Activated	2.985
SPDEF	Activated	3.046	HNF4A	Activated	2.937
AHR	Activated	3.022	ESRRA	Activated	2.852
CEBPA	Activated	2.972	CEBPA	Activated	2.244
TRIM24	Inhibited	−3.065	SREBF1	Inhibited	−5.146
NCOA3	Inhibited	−2.932	SREBF2	Inhibited	−4.227
HOXA10	Inhibited	−2.797	PPARGC1B	Inhibited	−3.614
FOXM1	Inhibited	−2.705	NRIP1	Inhibited	−3.187
E2f	Inhibited	−2.508	NFYA	Inhibited	−3.172
BCL6	Inhibited	−2.497	E2f	Inhibited	−2.795
TCF4	Inhibited	−2.312	Creb	Inhibited	−2.643
NRIP1	Inhibited	−2.262	ESR1	Inhibited	−2.621
MYC	Inhibited	−2.258	Nr1h	Inhibited	−2.59
MEF2D	Inhibited	−2.246	GATA3	Inhibited	−2.388

### Suppression of Wnt/β-catenin Signaling by *Scap* Deletion at E17.5

**Figure 6:**
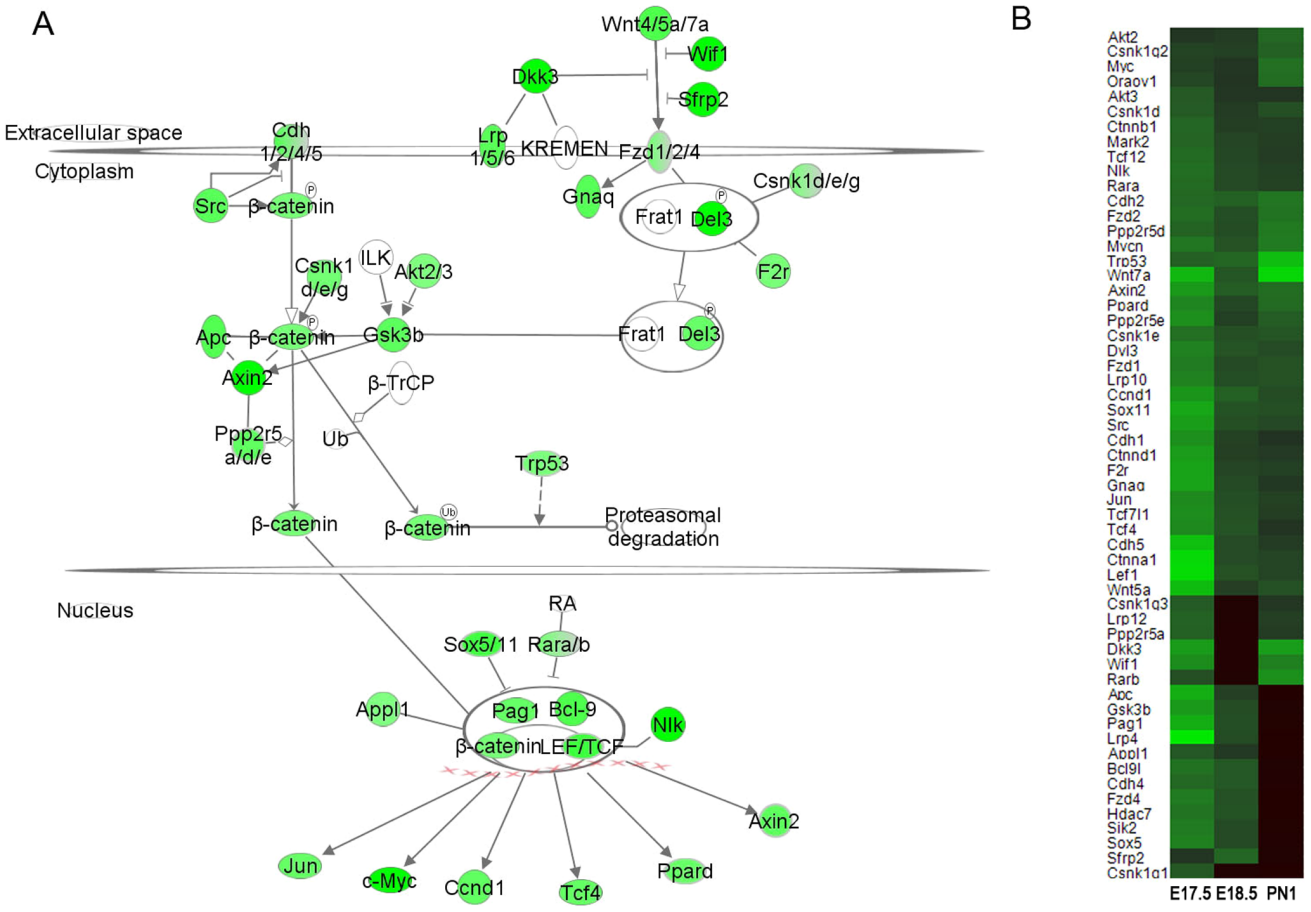
Epithelial *Scap* deletion in the perinatal lung suppresses Wnt/β-catenin signaling. (A) A schematic representation of mRNAs changed in the canonical Wnt/β-catenin pathway in *Scap^Δ/Δ^* perinatal lung (E17.5) is shown. Green nodes indicate mRNAs that were decreased in *Scap^Δ/Δ^* lungs with respect to control lungs. Wnt pathway genes that were not changed in response to *Scap* deletion are depicted as empty nodes. (B) Heatmap demonstrates decreased expression of genes encoding a number of key components in the Wnt/β-catenin pathway in *Scap^Δ/Δ^* vs. control mice, n = 3/group.

From the microarray analysis, a number of Wnt/β-catenin signaling components, including ligands (*Wnt4/5a/7a*), receptors (*Fzd1/2/4, Lrp 1/5/6, Cdh1/2/4/5*) and effectors (*Ctnnb1*) were decreased in *Scap^Δ/Δ^* mice, particularly at E17.5 (Figure 6). In addition, several factors known to regulate Wnt-induced transcription, including NEMO-like kinase (*Nlk*), *Sox5/11* and retinoic acid receptor (*Rara/b*), were also decreased in *Scap^Δ/Δ^* mice.

**Figure 7:**
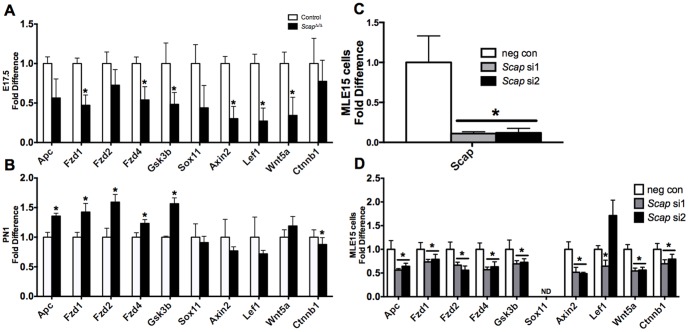
*Scap* modulates expression of Wnt pathway components. A and B) qPCR analysis demonstrates altered expression of Wnt signaling genes in *Scap^Δ/Δ^* E17.5 (A) and PN1 (B) whole lung tissue compared to littermate controls. Results were normalized to 18 s RNA, n = 3–4/group, *p<0.05 vs. control at the respective time point. C and D) qPCR analysis demonstrates decreased expression of *Scap* (C) and of Wnt pathway (D) genes in MLE15 cells following transfection with negative control (neg con) and two distinct *Scap* siRNAs (si1 and si2). Results were normalized to 18 s, n = 6 samples/group representing 3 independent experiments, *p<0.05 vs negative control. ND = non-detectable.

Suppression of the mRNAs of Wnt-pathway genes were confirmed by real time PCR analysis (Figure 7). Consistent with the RNA microarray findings, expression of several Wnt pathway genes were decreased in *Scap^Δ/Δ^* vs. control whole lung tissue at E17.5 (Figure 7A). Interestingly, the response of Wnt signaling genes in the absence of epithelial *Scap* were dynamic with respect to age. Expression of *Apc*, *Fzd1/2/4* and *Gsk3b* were slightly higher in *Scap^Δ/Δ^* vs. control lung tissue at PN1 (Figure 7B). We speculate that the suppression of Wnt pathway genes at E17.5 was due to the direct effect of *Scap* deletion from alveolar epithelial cells, while the moderate increase in a subset at PN1 may have resulted from the compensatory effect to the loss of SCAP in alveolar type II cells from adjacent cells such as lipofibroblasts, smooth muscle cells or endothelial cells [12].

To test if SCAP/SREBP signaling directly regulates Wnt pathway genes in pulmonary epithelial cells, we performed siRNA-mediated knockdown of *Scap* in an immortalized mouse lung epithelial cell line, MLE15 cells. This approach effectively reduced *Scap* mRNA expression to 10–20% of control levels (Figure 7C). Consistent with the microarray observation at E17.5, siRNA knockdown of *Scap* reduced expression of all the Wnt pathway genes tested except *Sox11* and *Lef1* (Figure 7D). Sox11 expression is undetectable in MLE15 cells, and results for Lef1 expression were inconsistent using two independent Scap siRNA duplexes. Collectively, *in vivo*, *in vitro* and *in silico* data support the concept that SCAP/SREBP signaling has stage-specific roles in the regulation of Wnt pathway genes during lung development and maturation.

### Suppression of Glucocorticoid Receptor Signaling by Epithelial *Scap* Deletion

Glucocorticoid receptor (GR) signaling plays important roles in many physiological processes, including lung development and maturation [23]. Cellular responses to glucocorticoids are mediated primarily through binding to the intracellular GR (NR3C1) [24] and translocation of this complex to the nucleus where it interacts with multiple transcription factors including JUN, Nuclear Factor-KappaB, Octamer transcription factors OCT1 and OCT2, CREB binding protein and STAT5 to positively and negatively modulate gene expression. Expression of multiple GR cofactors and target genes were altered in *Scap^Δ/Δ^* lungs with respect to controls at E17.5, E18.5 and PN1 (Table 5). The effect of SREBP on GR signaling was primarily seen at E17.5, where most of the GR-associated genes were decreased in perinatal *Scap^Δ/Δ^* lungs. Similar to the alterations observed in Wnt signaling, a subset of genes displayed a moderate increase of expression at PN1, which may be due to compensation via adjacent cells. Glucocorticoid receptor (*Nr3c1*), three GR co-activators *(Ncoa1, Ncoa2, and Ncoa3)* and GR target genes (*Tgfb1, Tgfb2 and Smad3*) were significantly decreased in *Scap^Δ/Δ^* lungs at E17.5 and E18.5. *Nr3c1* interacts with *Ncoa1* and *Ncoa2* to regulate transforming growth factor beta (*Tgfb1*) signaling via increased transcriptional activation of SMAD family member 3 (*Smad3*) [25]–[27]. *Ptgs2* was one of four genes linked to GR signaling that was significantly increased in perinatal *Scap^Δ/Δ^* lungs. In the rat renal cortex, *Ptgs2* expression is suppressed by endogenous glucocorticoid levels through *Nr3c1* and inhibition of *Nr3c1* results in increased expression of *Ptgs2* in the presence of dexamethasone [28]–[30]. These data indicate that epithelial *Scap/Srebp* signaling influences the expression of multiple GR signaling components in the lung in a time-dependent manner.

**Table 5:** Glucocorticoid receptor signaling was suppressed in *Scap* ^Δ/Δ^ mice in perinatal lung at E17.5.

Symbol	E17.5	E18.5	PN1	RefseqID
Foxo3	0.52	0.79	0.85	NM_019740
Polr2a	0.54	0.81	1.27	NM_009089
Map3k1	0.58	0.77	1.03	NM_011945
Nos2	0.60	0.79	0.68	NM_010927
Pik3r4	0.60	0.87	0.95	NM_001081309
Smad3	0.61	0.80	0.93	NM_016769
Tgfb2	0.62	0.77	0.85	NM_009367
Pou2f1	0.63	0.77	1.52	NM_011137
Polr2b	0.66	0.90	0.73	NM_153798
Smarca2	0.66	0.90	1.01	NM_011416
Smarca4	0.66	0.77	1.30	NM_001174078
Crebbp	0.66	0.79	1.82	NM_001025432
Nrip1	0.67	0.87	1.19	NM_173440
Jun	0.67	0.82	0.89	NM_010591
Atm	0.67	0.90	1.19	NM_007499
Nfatc3	0.67	0.83	0.75	NM_010901
Ncoa1	0.68	0.85	1.24	NM_010881
Sos1	0.68	0.90	0.91	NM_009231
Ncor2	0.68	0.78	1.41	NM_011424
Nfkb1	0.69	0.92	0.84	NM_008689
Pik3r1	0.69	0.84	1.38	NM_001077495
Ncoa3	0.70	0.88	1.10	NM_008679
Cdk7	0.71	0.90	0.58	NM_009874
Nr3c1	0.72	0.87	0.97	NM_008173
Ncoa2	0.76	0.86	1.43	NM_008678
Mapk14	0.80	0.89	0.87	NM_011951
Mapk3	0.81	0.89	0.68	NM_011952
Cebpa	0.82	0.91	0.48	NM_007678
Stat5b	0.84	0.88	0.71	NM_001113563
Mapk10	0.91	1.13	0.68	NM_009158
Pik3c2g	1.15	1.32	1.83	NM_207683
Polr2k	1.40	1.84	1.30	NM_001039368
Ptgs2	1.98	1.72	0.50	NM_011198

### miRNAs Responsive to *Scap* Deletion in Perinatal Lung

MicroRNAs control many physiological and pathological processes through post-transcriptional regulation of gene expression. To assess the role of the SREBP signaling cascade in the regulation of miRNAs, genome-wide miRNA expression profiling was compared in *Scap^Δ/Δ^* and control mice at E18.5. We identified 22 miRNAs that were differentially expressed in response to *Scap* deletion in epithelial cells (Table 6). Among these, 11 miRNAs were increased and 11 were decreased. Target genes were predicted for differentially expressed miRNAs using the miRNA Target Filter tool in Ingenuity Pathway Analysis. Target genes were further filtered if their differential expression patterns were negatively correlated to those of differentially expressed miRNAs in *Scap^Δ/Δ^* vs. control mice. Several miRs associated with the regulation of lipid metabolism were differentially expressed in *Scap^Δ/Δ^* lungs at E18.5, including miR-122, miR-33, miR-29, miR-106 and miR-335 (Table 6). These data suggest that, in addition to the transcriptional regulation of lipid homeostasis by SREBPs, altered miRNAs in response to Scap/Srebp deletion is likely to further modulate lipogenic gene expression at the posttranscriptional level to regulate lipid homeostasis.

**Table 6:** Differentially expressed miRNA in E18.5 *Scap* ^Δ/Δ^ vs. control mouse lungs.

Symbol	Fold Change	Target Count	Function and Reference
miR-122	2.5	17	key regulator of cholesterol and fatty-acid metabolism (Esau, 2006); regulate lipogenic gene expression including SREBF-1c, Fasn, Hmgcs1, Sqle and Acaca (Krutzfeldt, 2005; Iliopoulos, 2010)
miR-196a	1.79	23	inducible brown adipogenesis by suppresse the expression of the white-fat gene Hoxc8 (Mori, 2012)
miR-602	1.75	10	
miR-449c	1.72	51	highly expressed in lung shortly before and after birth, induce epithelia differentiation by repressing the Notch pathway (Bao, 2012, Lize, 2010)
miR-1202	1.64	27	
mir-4784	1.64	4	
miR-647	1.56	32	
miR-29a	1.54	21	pathogenesis of pulmonary fibrosis (Cushing, 2010), regulates pro-inflammatory cytokine secretion by targeting Insig1, Srebf1 and Lpl (Chen, 2011,Hulsmans 2011)
miR-192	1.54	1	
miR-15a	1.52	8	pre-adipocyte differentiation, targeting DLK1(McGregor, 2011)
miR-375	1.28	7	alveolar epithelial cell differentiation by inhibiting Wnt/β-catenin pathway (Wang, 2012)
miR-103a	−1.3	15	regulation of lipid metabolism (Wilfred, 2007)
miR-33	−1.35	8	key post-transcriptional regulator of cellular cholesterol and lipid homeostasis, hosted within the introns of Srebf2 and Srebf1 (Rayner, 2010; Marquart, 2010; Najafi-Shoushtari, 2010)
miR-335	−1.37	2	up-regulation of microRNA-335 is associated with lipid metabolism and obesity (Nakanishi, 2009)
miR-363	−1.54	22	
miR-185-5p	−1.57	17	regulates adipocyte differentiation, up-regulated in mature adipocytes while down-regulated in obese men (Ortega, 2010)
miR-376b	−1.58	26	
miR-204	−1.62	29	regulated by STAT3, down-regulated in pulmonary arterial hypertension(Courboulin, 2011)
miR-1	−1.66	29	regulate by SREBF1 and Cebpa (Granjon, 2009)
miR-290-5p	−1.8	12	
miR-124-3p	−1.86	43	down-regulated during fetal lung development, inhibits fetal alveolar epithelial cell maturation
miR-133b	−1.91	25	regulate by SREBF1 (Granjon, 2009), inflammatory lung disease(Oglesby, 2010); insulin-dependent diabetes mellitus (Li, 2009)

Several miRNAs known to influence lung development, maturation and disease were also differentially expressed in *Scap^Δ/Δ^* vs. control lungs including miR-1, miR-124, miR-29 and miR-133. Previous data demonstrated a link between miR-1 and glucocorticoid signaling. Treatment with dexamethasone and myostatin induced miR-1 expression via nuclear translocation of *Nr3c1* [31]. miR-1 is known to be regulated by *Srebf1* and *Cebpa*, both of which are important regulators of lipogenesis in alveolar type II cells [32]. miR29 is associated with the pathogenesis of pulmonary fibrosis [33] and regulates pro-inflammatory cytokine secretion by targeting *Insig1*, *Srebf1* and *Lpl* [34], [35]. miR-133, regulated by SREBP1 [32], is associated with inflammatory lung disease [36]. Our data support the concept that a group of microRNA involved in lipid metabolism and lung development/maturation were dysregulated in response to *Scap* deletion in epithelial cells, potentially contributing to the crosstalk between SREBP signaling and pathways critical for lung development and maturation (e.g., glucocorticoid signaling pathway), connecting the “lipid” and “lung” related transcripts within the SREBP network.

## Discussion

We sought to identify the role of epithelial SREBP, a known regulator of cellular lipid homeostasis in multiple tissues, during a critical time period of perinatal lung development and maturation from E17.5 to PN1. Our data show that SCAP/INSIG/SREBP signaling primarily influences epithelial development, cell death and cell proliferation at E17.5, while primarily influencing surfactant physiology, lipid/sterol synthesis, and phospholipid transport at PN1. In addition to regulating the lipogenesis program that was dominant at the later stage of lung development, this study demonstrates cross-talk between SREBP signaling and two other signaling pathways important for lung development and maturation, Wnt/β-catenin and glucocorticoid receptor signaling. Epithelial SCAP/INSIG/SREBP signaling regulates expression of miRNAs that control lipid homeostasis and glucocorticoid signaling at the post-transcriptional level. Taken together, our analysis demonstrates that SCAP/INSIG/SREBP regulates perinatal lung lipogenesis and maturation through its interactions with diverse regulatory partners.

SatPC pool sizes were significantly decreased in *Scap^Δ/Δ^* lungs at E18.5 and PN1 (Figure 2A). SatPC production and secretion by alveolar type II cells increases prior to birth to facilitate the transition to air breathing. Synthesis of SatPC occurs by direct de novo synthesis (Kennedy pathway) and the remodeling of existing monosaturated PC species in alveolar type II cells (Lands cycle, also known as the remodeling pathway); the relative contribution of these pathways to the total SatPC pool is approximately equal *in vitro* and *in vivo Pcyt1a* is the rate-limiting enzyme for the de novo synthesis pathway and is critically required for SatPC production and lung function at birth [37]. Two genes in the de novo synthesis pathway (*Pcyt1a and Pld4*) and two in the remodeling pathway (*Lpcat1 and 3*) were significantly decreased in perinatal *Scap^Δ/Δ^* lungs, all four of which belong *to* the lipid-enriched cluster, Cluster 5 (Figure 4B, Table S1). *Lpcat1* and *Lpcat3* encode lysophospholipid acyltransferases that, in conjunction with phospholipases, generate SatPC via the remodeling pathway. *Lpcat1* primarily reacylates lysoPC with a saturated fatty acid donor and is required for pulmonary SatPC production in fetal mice [38]. *Lpcat3* also demonstrates lysoPC acyltransferase activity, is expressed in the lung and at high levels in the liver where it drives phospholipid remodeling [39], [40]. *Pcyt1a*, *Lpcat1 and Pld4* have putative Srebp binding sites in their proximal promoters that are conserved between mouse, rat and human, suggesting that they are likely direct targets of the SCAP/SREBP/INSIG pathway (data not shown). Collectively, in addition to driving the initial phases of lipogenesis in the lung (discussed below), these data are consistent with the concept that *Scap* also drives the final steps of SatPC synthesis during late pulmonary maturation, in part, through induction of *Pcyt1a*, *Lpcat1*, *Lpcat3* and *Pld4* expression.

We used a combination of profile clustering, promoter mining and literature mining approaches to identify potential upstream regulators and downstream targets of SCAP. Through this analysis, differentially expressed genes in *Scap^Δ/Δ^* vs. control mice were placed into 8 distinct clusters based on expression similarity. Genes in clusters 5 and 7 were both functionally involved in the lipid synthesis, metabolism and transport. Additionally, cluster 7 genes also play important roles in lung development and function including *Sftpb, Abca3, Fgf18 and Pdgfa*. Mutations in the genes encoding *Sftpb* and *Abca3* are associated with respiratory distress and interstitial lung disease in the pediatric population (SFTPB; Online Mendelian Inheritance in Man [OMIM] number 178640, ABCA3; OMIM number 601615) and mutations in the human SFTPA1 gene (c.655C>T (rs4253527)) increase the risk of idiopathic pulmonary fibrosis (http://www.ncbi.nlm.nih.gov/clinvar/RCV000014093.1/). *Fgf18* is required for late stage embryonic lung alveolar development as *Fgf18* deficiency in mice resulted in reduced alveolar spaces associated with thickened interstitial mesenchymal compartments [41]. *Pdgfa* is critical for alveolar myofibroblast ontogeny. Postnatally surviving *Pdgfa(−/−)* mice develop emphysema secondary to the failure of alveolar septation [42]. Other notable genes in cluster 7 include *Hes1*, *Hyal1*, *Clic5*, *Cdo1*, *Hck*, and *Twist2*, the deletion/mutation of which all lead to abnormal pulmonary alveolar morphology (information from http://www.informatics.jax.org/phenotypes.shtml MP:0002132).

Potential upstream regulator analysis of cluster 5 (C5) and cluster 7 (C7) genes identified SREBP as a common key regulator for both C5 and C7 genes; however, other potential key regulatory partners in C5 and C7 were distinct. As shown in Figure 4B, *Hnf4a*, *Pax3*, and *Nfe2l2* were predicted as major regulators working in concert with SCAP/INSIG/SREBP signaling to control expression of C5 genes. *Hnf4* (Hepatocyte nuclear factor 4-alpha) was ranked as the 6th important key regulatory hub of C5 based on over-representative p-values (SREBP2, SCAP, SREBP1, INSIG1, INSIG2 were listed 1–5) and as the top TF in the network based on the target gene numbers in C5 (regulating 15% of C5 genes, data not shown). HNF4 regulates a number of genes involved in fatty acid synthesis (*Acly*, *Asah1*, *Mid1ip1*, *Scd1* and *Tp53*); phospholipid synthesis (*Acp6*, *Pcyt1a*, *Lypla2*, *Pitpnb* and *Scd1*); and lipid transport (*Slc25a1*, *Slc17a5*, *Rab7a*, and *Asah1*). Multiple lines of evidence suggest that SREBP1/2 directly binds and regulates *Hnf4* expression *in vivo* and *in vitro* [43]–[46]. *Nfe2l2* (Nuclear factor erythroid 2-related factor 2) and *Pax3* (paired box gene 3) influence *Srebf1* expression in mouse liver and human medulloblastoma cell line [47], [48]. On the other hand, *Por, Pparg, Cebpa, Nkx2-1, Tgfb1, Ctnnb1, Foxo1, Foxa2, and Ifng* were predicted as central hubs working together with SREBP to regulate gene expression in cluster 7 (Figure 4C). *Srebf1*, *Cebpa* and *Pparg* are known transcription factors that regulate lipogenesis in various tissues. CEBPA directly binds to the promoter of *Srebp-1c* and positively regulates SREBP1c expression during adipogenesis [49]. Activation or deletion of SREBP1c lead to a corresponding increase or decrease in CEBPA expression in hepatocytes and adipocytes [49]–[51]. Similarly, SREBP1c and PPARG transactivate each other's expression in hepatic cells and adipocytes [52]–[55], therefore SREBP, CEBPA and PPARG share the same transcriptional network controlling lipogenesis and metabolism. *Foxo1* is a master regulator of energy metabolism in multiple tissues [56]. In the liver, *Cebpa* forms an interaction complex with *Foxo1* to suppress *Foxo1* expression [57], [58] and *Foxo1* inhibits SREBP-1c expression and other lipogenic genes [59]. Previous work has shown that NKX2-1 and SREBP co-regulated *Abca3* expression through two distinct cis-acting regulatory cassettes, a ‘lung regulatory unit’ (TTF-1, GATA, FOXA2, CEBPA, etc.) and a ‘lipid regulatory unit’ (SREBP, SP1) present in *Abca3* promoter [60]. It is likely that genes sharing both ‘lung’ and ‘lipid’ properties in cluster 7 are regulated through similar mechanisms.

One unexpected finding from this study was suppression of multiple Wnt pathway components in the *Scap^Δ/Δ^* lungs, including ligands (*Wnt 5/5a/7a*), receptors (*Fzd 1/2/4*) and effectors (*Gsk3b, Ctnnb1*) (Figure 6, Figure 7A and 7B). Results initially identified by microarray analysis were confirmed at the mRNA level in MLE15 cells in which endogenous *Scap* was inhibited, demonstrating a direct effect of *Scap* in the regulation of specific Wnt signaling pathway components (Figure 7D). Wnt signaling is critically required for pulmonary epithelial and vascular development [61]–[64] and has been implicated in chronic lung diseases including pulmonary fibrosis [65]–[67] and chronic obstructive pulmonary disease [68], [69]. Defects in lung morphology were not observed in *Scap^Δ/Δ^* lungs in the present study (data not shown). One possible explanation is that the degree of Wnt suppression in *Scap^Δ/Δ^* lungs was insufficient to phenocopy mouse models of epithelial Wnt loss [70], [71]. It is also possible that residual *Scap* expression, due to incomplete deletion of *Scap* from epithelial cells in our model, was sufficient to maintain Wnt signaling adequate to support normal pattering of the lung.

In addition to its role in organogenesis, the Wnt signaling pathway has emerged as a key regulator of lipogenesis in mice and humans where it acts as a suppressor of adipocyte maturation. Three Wnt ligands have been implicated in suppression of adipogenesis in mice including WNT10b, WNT5a and WNT5b [72]. Enhanced Wnt signaling blocks adipogenesis via inhibition of the central transcriptional regulators CEBPA and PPARg [73]. The biological significance of Wnt suppression in *Scap^Δ/Δ^* lungs is currently unknown. However, we postulate that suppression of Wnt signaling in *Scap^Δ/Δ^* alveolar type II cells serves as a compensatory mechanism to increase surfactant lipogenesis in the absence of SCAP/INSIG/SREBP signaling. The compensatory regulation of lipogenesis in the absence of SCAP in epithelial cells is consistent with previous findings in the adult lung wherein phospholipid synthesis was decreased in type II cells of adult *Scap^Δ/Δ^* mice; storage, synthesis, and transfer of lipids by lung lipofibroblasts were increased [12]. Similarly, SCAP deletion in the liver resulted in a compensatory increase in fatty acid synthesis in adipocytes [74].

MicroRNAs have emerged as key regulators of various cellular metabolic pathways, including lipogenesis. In this study we identified multiple miRs that were differentially expressed in *Scap^Δ/Δ^* mice. miR122 and miR33 are known to directly regulate lipid metabolism and lipid homeostasis in the liver [75], [76]. miR-33 was identified as a key post-transcriptional regulator of cellular cholesterol homeostasis and is present in several animal species, including humans. Humans possess two family members called mir-33a and mir-33b, which are located within intron 16 of *Srebf2* and intron 17 of *Srebf1*, respectively. Both mir-33a and mir-33b are co-transcribed with their host genes (*Srebf1/2*) and reciprocally regulate genes involved in cholesterol and fatty acid metabolism [77]–[79]. Probes targeting multiple species of miR-33 were decreased in *Scap^Δ/Δ^* vs. control mice. miR-122 is highly conserved from human to frogs and most abundantly expressed in liver. Blockade of miR-122 expression decreased *Srebf-1c*, *Fasn*, *Hmgcs1*, *Sqle* and *Acaca* expression, resulting in the reduction of plasma cholesterol and triglyceride levels in both rodents and primates [80], [81]. The fact that miR-122 expression correlated with *Srebf1* and other lipid metabolism related genes indicated that these genes are not direct targets of miR-122. The mechanism by which miR122 activates these genes and lipid metabolism is currently unknown. miR122 was induced 2.5 fold in *Scap^Δ/Δ^* vs. control mice, perhaps serving as a compensatory response to modulate SREBP and other lipogenic genes. Other miRNAs involved in regulating lipid homeostasis including miR-29 [82], miR-106 [83], and miR-335 [84] were differentially expressed in *Scap^Δ/Δ^* vs. control mice. Our data suggest that in addition to the transcriptional regulation of lipid homeostasis by SREBPs, miRNAs may play important roles at the posttranscriptional level to influence lipogenic gene expression and maintain lipid homeostasis.

In summary, our previous network model identified the important role of SREBP as a regulatory hub controlling a lung lipid transcriptional network [14]. The present study was designed to further define the dynamic roles of SREBP during perinatal lung development and maturation. SREBP signaling influences genes controlling epithelial development, cell death and cell proliferation at E17.5 and those involved in surfactant physiology, lipid/sterol synthesis, and phospholipid transport at a later gestational age (PN1). Our study supports the concept that SREBP signaling interacts with other signaling pathways important for lung development and maturation, including the Wnt/β-catenin pathway. SREBP regulates perinatal lung lipid homeostasis and lung maturation through multiple mechanisms including its interactions with diverse regulatory partners to regulate target genes and microRNAs that are known to regulate lipid metabolism at the posttranscriptional level.

## Supporting Information

Table S1 Genes differentially expressed in Scap^Δ/Δ^ mice during perinatal lung maturation.
(XLSX)

Table S2 Comparison of genes differentially expressed in Scap^Δ/Δ^ and/or in Insig1/2^Δ/Δ^ mice.
(XLSX)
